# Multiplex detection of ten *ESR1* mutations and *AKT1* E17K in breast cancer using digital PCR

**DOI:** 10.1016/j.jlb.2024.100154

**Published:** 2024-03-30

**Authors:** Stavroula Smilkou, Aliki Ntzifa, Dimitra Stergiopoulou, Vasilis Georgoulias, Evi Lianidou

**Affiliations:** a Analysis of Circulating Tumor Cells, Laboratory of Analytical Chemistry, Department of Chemistry, University of Athens, 15771, Athens, Greece; b First Department of Medical Oncology, Metropolitan General Hospital, Athens, 15562, Greece

**Keywords:** Estrogen receptor, Breast cancer, *ESR1* mutations, Crystal digital PCR, Liquid biopsy, Plasma-cfDNA

## Abstract

**Introduction:**

*ESR1* mutations are now established as a key mechanism of resistance to endocrine therapy in estrogen-receptor–positive breast cancer (ER​+ ​breast cancer) and their sensitive and specific detection in plasma-cell free DNA (plasma-cfDNA) is crucial to monitor during patient treatment. In the present proof-of-principle study, we evaluated the performance of a novel multiplex assay (12plex) for the detection of ten *ESR1* mutations and *AKT1* E17K in plasma-cfDNA based on Crystal Digital PCR® (Stilla Technologies, France).

**Materials & methods:**

We analyzed 35 plasma-cfDNA samples from ER+ ​breast cancer patients and 10 samples from healthy donors and further compared the results with our previously reported *ESR1* NAPA assay for D538G, Y537S, Y537C and Y537 N *ESR1* mutations.

**Results:**

Using this novel 12plex *ESR1-AKT* 6-color Crystal Digital PCR® assay we detected both *AKT1* E17K and *ESR1* D538G mutations in 5/35 (14.3%) plasma-cfDNA samples. *ESR1* D538G was detected in 4/35 (11.4%) of these plasma-cfDNA samples using the *ESR1* NAPA assay. Direct comparison between Crystal Digital PCR™ and the *ESR1* NAPA assay revealed a high concordance (97.1%, k = 0.871, p < 0.001) for the detection of D538G mutation.

**Conclusion:**

The Stilla 12plex *ESR1-AKT* 6-color Crystal Digital PCR® assay is multiplex, highly sensitive and robust and can be used in liquid biopsy.

## Highlights

• The 12plex *ESR1-AKT* 6-color Crystal Digital PCR® assay was designed for the simultaneous detection in a single reaction of ten *ESR1* mutations and *AKT1* E17K in liquid biopsy samples.
• Using plasma-cfDNA analysis to track *ESR1* mutations is highly beneficial for identifying tumor molecular dynamics and improving personalized treatments for mBC patients.
• Highly multiplexed Crystal Digital PCR® has demonstrated significant potential for clinical applications in monitoring ER ​+ ​breast cancer patients.
• The Stilla *ESR1-AKT* 6-color Crystal Digital PCR® assay is quantitative, highly sensitive and easy to perform in plasma-cfDNA samples.

## Abbreviations

ARMS: Amplification-Refractory Mutation System
*AKT1*: AKT serine/Threonine Kinase 1
BC: Breast Cancer
cfDNA: cell-free DNA
CTCs: Circulating Tumor Cells
ctDNA: circulating tumor DNA
*ESR1*: Estrogen Receptor 1
ET: Endocrine Therapy
gDNA: genomic DNA
LB: Liquid Biopsy
MAF: Mutation Allele Frequency
NaME-PrO: Nuclease-assisted Minor Allele Enrichment using Overlapping probes
SERD: Selective Estrogen Receptor Degrader

## 1 Introduction

Liquid biopsy (LB) is a minimally invasive tool that involves the analysis of peripheral blood and has proven to be effective in monitoring treatment outcomes for a wide range of cancer types [[1], [2], [3]]. LB analysis includes the enumeration and molecular characterization of circulating tumor cells (CTCs), and the detection of circulating tumor DNA (ctDNA) in plasma cell-free DNA (plasma-cfDNA), circulating cell-free microRNAs (ccfmiRNAs), and extracellular vesicles (EVs) that are shed from primary tumors and their metastatic sites into the blood circulation [4]. Recently, liquid biopsy involves the analysis of biomarkers in other biological fluids as well. Numerous clinical studies have demonstrated that ctDNA analysis in plasma-cfDNA can predict disease progression before clinical confirmation, uncover therapeutic resistance, track treatment response, and find actionable genetic abnormalities [5]. Standardization of assays for plasma-cfDNA testing and pre-analytical conditions is highly important for the proper implementation of ctDNA testing [6].

Breast cancer is a very heterogeneous and dynamic disease with unique somatic changes leading to disease recurrence and resistance. Breast cancer survival rates vary across the world due to several reasons, such as delayed diagnosis and inadequate access to effective treatments [7]. The most frequent breast cancer type is the hormone receptor-positive (HR+), which expresses the estrogen receptor (ER) and/or progesterone receptor (PR) and accounts for approximately 75% of BC cases [8]. The development of resistance to endocrine therapy (ET) is a crucial step in the progression of ER​+ ​breast cancer, since it is linked to a greater likelihood of recurrence and increased mortality rates [9]. *ESR1* mutations have been recently shown to be a key mechanism of resistance to ET in ER+ ​breast cancer, making the detection of these mutations critical for monitoring patient treatment [10]. In the past decade, LB analysis has been employed in multiple clinical trials to investigate the prevalence of *ESR1* mutations in ER+ ​breast cancer [[11], [12], [13]]. Highly sensitive and specific detection of *ESR1* mutations in LB analytes as plasma-cfDNA and/or CTCs is highly important for patient/treatment selection and monitoring of ET efficacy [14]. On January 27, 2023, the Food and Drug Administration (FDA) approved elacestrant (Orserdu, Stemline Therapeutics, Inc.) for postmenopausal women or adult men with ER+, HER2-, *ESR1*-mutated advanced or metastatic breast cancer with disease progression following at least one line of endocrine therapy. In parallel, the FDA approved the Guardant360 CDx blood test as a companion diagnostic for *ESR1*-mutated ER+ ​advanced or metastatic breast cancer patients who have disease progression following at least one line of ET and may benefit from elacestrant treatment [15,16]. Additionally, in ER+ ​metastatic breast cancer patients, *AKT1* E17K mutation is detected at frequencies between 1.4% and 8.2%, with a mean mutation frequency of 3.8% [17]. It has been recently reported that the duration of mTOR inhibitor therapy was longer in *AKT1*-mutant cases, but this was not identified in clinical trials previously due to the rarity of this alteration, and overall survival (OS) was similar between *AKT1*-mutant cases and *AKT1*-wildtype controls [18].

Highly sensitive detection methods for the genotyping of tumors, such as next-generation sequencing (NGS) or droplet digital PCR (ddPCR), are major players in LB analysis, since they play an important role in the application of targeted therapies for treating cancer patients [19]. Up to now there is a plethora of applications of digital PCR technologies in liquid biopsy [[20], [21], [22], [23], [24]]. Crystal Digital PCR®, is based on the partition of the sample into a 2D array of thousands of individual droplet reaction compartments that individually amplify nucleic acid molecules providing high sensitivity and enhancing low-level detection of multiple reactions in parallel. In this technology, up to six different fluorescent light channels are used, and different fluorophores tag individual reactions. Based on a combination of these fluorescent tags, multiplex detection of multiple target mutations can be achieved, leading to a significant decrease of assay cost and analysis time. Furthermore, high multiplexing digital PCR enables multiple controls to be included in a single assay for increased data reliability.

Our group has recently developed a highly sensitive and specific assay for the detection of *ESR1* mutations in plasma-cfDNA. This assay, called *ESR1*-NAPA (NaME-PrO-assisted ARMS) successfully resolves false positive signals generated during classic ARMS-PCR and is based on the combination of two steps: a step called NaME-PrO (Nuclease-assisted Minor Allele Enrichment using Overlapping probes) that is used to remove the wild type DNA (wtDNA), followed by an ARMS-PCR step to detect four *ESR1* mutations individually using real-time PCR-melting curve analysis [25]. NaME-PrO is used to remove wild type DNA from multiple DNA targets before DNA amplification. This method ensures that the downstream genomic analysis processes are left substantially unchanged, while providing the benefit of minimizing interference from any “unwanted” DNA [26].

The aim of the present proof-of-principle study was to evaluate the performance of a novel multiplex crystal digital PCR assay (12plex *ESR1-AKT* 6-color Crystal Digital PCR® assay Stilla Technologies, France), for the simultaneous detection in a single reaction of ten *ESR1* mutations and *AKT1* E17K in plasma-cfDNA samples from patients with ER​+ ​breast cancer. Towards this, we analyzed in parallel identical plasma-cfDNA samples for *ESR1* D538G, Y537S, Y537C and Y537N mutations with the multiplex Crystal Digital PCR® assay and the *ESR1*-NAPA assay and compared directly the results.

## 2 Materials and methods

### 2.1 Clinical samples

We analyzed 35 plasma-cfDNA samples obtained from nine ER+ ​breast cancer patients with verified metastasis, receiving hormone therapy, at different time points under treatment during a 5-year follow-up period and ten healthy donors (HD). These samples were previously analyzed for *ESR1* mutations, using the *ESR1*-NAPA assay [25]. The study participants provided their informed consent by signing a form, and the ethics and scientific committees of our institutions approved the experimental protocol. The plasma was obtained by centrifugation of 10 mL PB (in EDTA) at 530g for 10min at room temperature, and a second centrifugation at 2000*g* for 10min, transferred into clean 2 mL tubes, and stored at −70°C. Plasma-cfDNA was isolated using the QIAamp Circulating Nucleic Acid Kit (Qiagen, Hilden, Germany), as previously described [25]. The final elution volume of extracted cfDNA was 50 μL.

### 2.2 Quality control of plasma-cfDNA

Quality control was performed to evaluate the concentration of plasma-cfDNA by using the Stilla LBx QC-assay. This assay allows with one single dPCR test to: (i) precisely titer the concentration of the wild-type cfDNA, (ii) evaluate the fragmentation size and quality of the sample, (iii) detect and quantify the “contamination” with human genomic DNA (hgDNA) that increases the concentration of wild-type DNA and introduces a bias in the measurement of the Mutation Allele Frequency, MAF(%), which can be very critical for longitudinal studies, and (iv) measure the extraction yield, for various fragment lengths, in order to properly evaluate the initial concentrations and the MAF(%) in plasma. The amount of plasma-cfDNA to be analyzed with the *ESR1-AKT* 6-color Crystal Digital PCR® assay was calculated based on its concentration measured with the LBx QC assay and could not exceed 5 ng of DNA per reaction.

### 2.3 Crystal digital PCR®

**Fig. 1:**
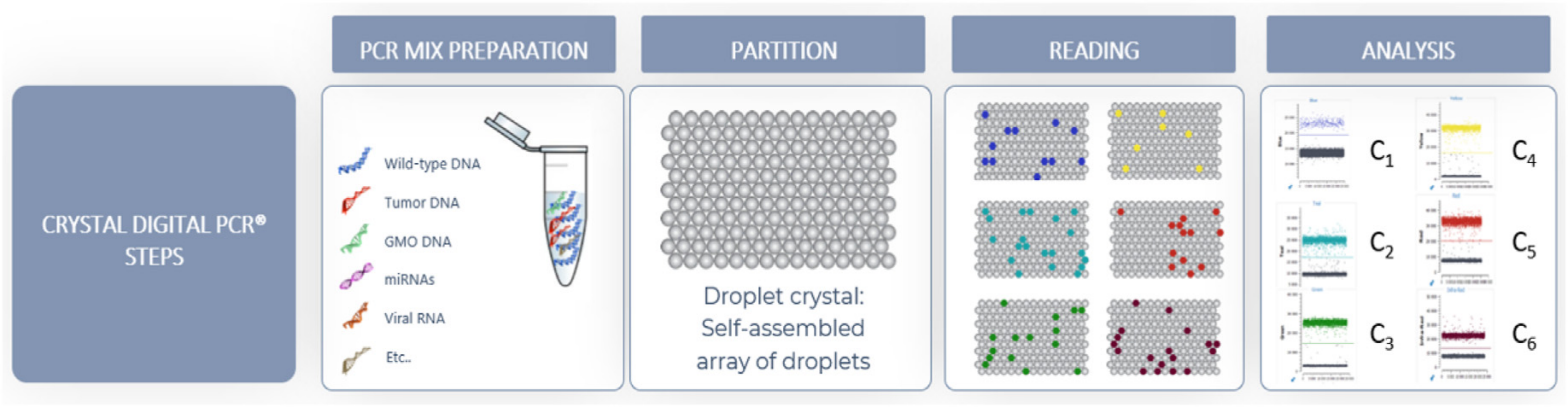
Workflow for crystal digital PCR®.

The Stilla 12plex *ESR1-AKT* 6-color Crystal Digital PCR® assay allows the simultaneous detection in the same sample of twelve gene targets: ten most prevalent *ESR1* mutations, *AKT1* E17K mutation and translin (*TSN*), used as a housekeeping gene. The assay is using the appropriate sets of primers and Taqman hydrolysis probes and the naica® multiplex PCR MIX 10X (Stilla Technologies, Villejuif, France) in a single Sapphire reaction (Fig. 1). The entire process occurs within the Sapphire microfluidic chip. The system requires the use of two instruments: i) the naica Geode, which combines sample partitioning and thermocycling of the droplet crystals and ii) the naica Prism6, an automated fluorescence microscope equipped with 6 distinct fluorescence channels (blue, teal, green, yellow, red and infrared) (Fig. 1).

**Fig. 2:**
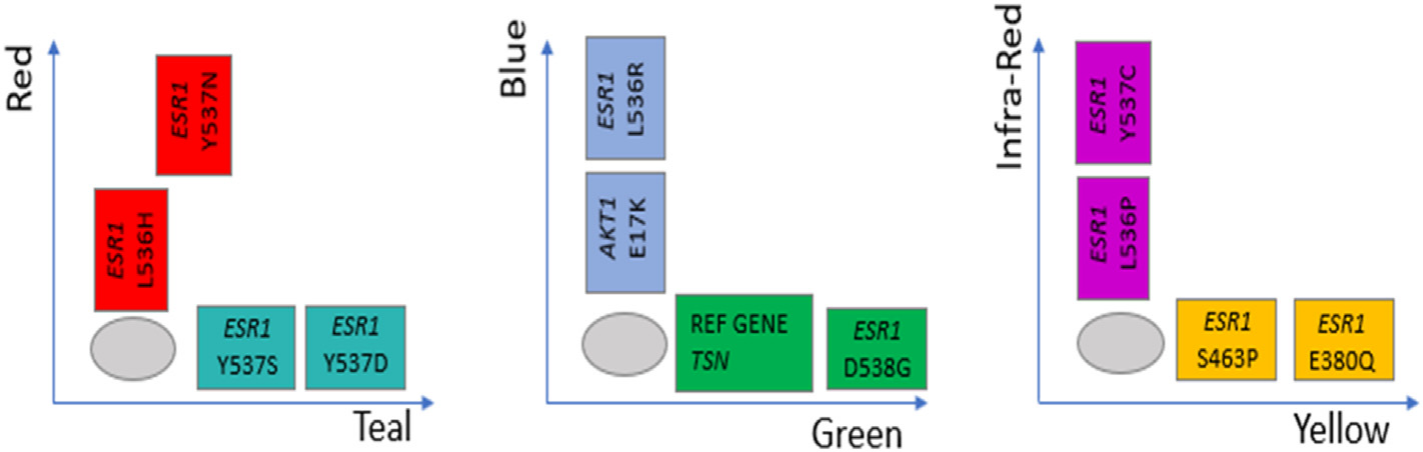
Stilla *ESR1-AKT* 6-color Crystal Digital PCR® assay: schematic of positive and negative populations positions on Crystal Miner 2D-dotplots. (For interpretation of the references to color in this figure legend, the reader is referred to the Web version of this article.)

All samples were processed with Crystal Digital PCR® on the 6-color naica® system using the Stilla LBx QC assay and the *ESR1-AKT* 6-color Crystal Digital PCR® assay, according to the instructions provided by the manufacturer (Fig. 2).

**Fig. 3:**
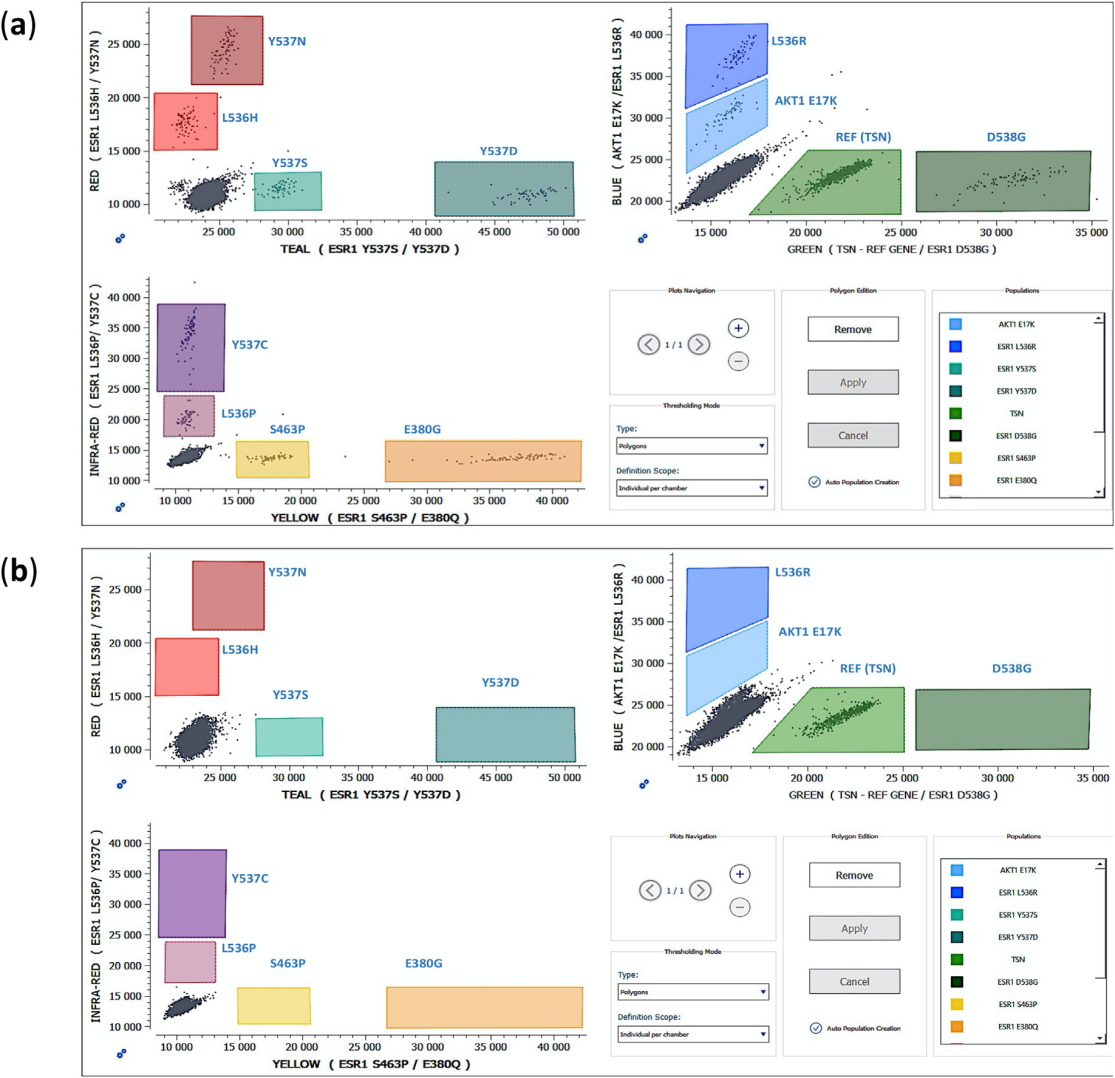
Characteristic 2D plots, illustrating the placement of the polygons of: (**a**) the positive control for *ESR1-AKT* 6-color Crystal Digital PCR® assay using the Crystal Miner software; (**b**) one WT plasma-cfDNA sample. (For interpretation of the references to color in this figure legend, the reader is referred to the Web version of this article.)

The Stilla 12plex *ESR1-AKT* 6-color Crystal Digital PCR® assay was designed and validated using reference materials purchased from commercial providers: mutated synthetic DNA (gBlocks™, IDT, Coralville Iowa USA) (Fig. 3a) and WT human genomic DNA (ENZ-GEN117-0100, Enzo Life Sciences, Farmingdale New York USA) (Fig. 3b).

In order to evaluate the analytical sensitivity of the multiplex assay for each of *ESR1* and *AKT1* mutations, the Limit of Blank (LoB) and the Limit of Detection (LoD) were determined following the Clinical and Laboratory Standards Institute (CLSI) EP17-A2 standard (Protocols for Determination Of Limits Of Detection And Limits Of Quantitation; Approved Guideline) LoB and LoD characterization method is described online [27]. Specificity was validated using WT human genomic DNA and mutant synthetic DNA. MAF (%) of mutated samples were calculated based on the concentration of target X (copies/μL), referred as C[X]. In accordance with the concentration of total referred as C [DNA tot] *(1)*.

(1) C [DNA tot] = [TSN-Ref gene] concentration (copies/μL)

As a result, MAF (%) in each positive mutation of a plasma-cfDNA sample was calculated based on the equation (2):

(2) MAF [X] ​= ​C[X]∗100/C [DNA tot]

### 2.4 Statistical analysis

The statistical analysis was conducted using IBM SPSS Statistics version 28.0 (SPSS Inc., Chicago, IL, USA).

## 3 Results

The LoBs and LoDs for each one of the 11 mutations tested are displayed in Table 1. The LoB refers to the upper limit target concentration considered acceptable in a blank sample. The LoD refers to the minimum detectable concentration of target molecules. LoB and LoD values are concentrations in the Sapphire chip chamber, measured in copies per microliter (copies/μL).

**Table 1:** *ESR1* and *AKT1* gene mutations detected by the Stilla *ESR1-AKT* 6-color Crystal Digital PCR® assay.

Gene	Mutation	Base changes	Cosmic ID	LoB (copies/μL) n = 30	LoD (copies/μL) n = 30
***TSN***	Reference gene	*Not Applicable*	*Not Applicable*	*Not Applicable*	*Not Applicable*
***ESR1***	**E380Q**	c.1138G > C	COSM3829320	0.00	0.20
	**S463P**	c.1387T > C	COSM4771561	0.14	0.43
	**L536H**	c.1607T > A	COSM6843697	0.27	0.62
	**L536P**	c.1607T > C	COSM6906109	0.14	0.43
	**L536R**	c.1607T > G	COSM4774826	0.00	0.20
	**Y537D**	c.1609T > G	COSM6918757	0.00	0.20
	**Y537N**	c.1609T > A	COSM1074635	0.14	0.43
	**Y537S**	c.1610A > C	COSM1074639	0.21	0.53
	**Y537C**	c.1610A > G	COSM1074637	0.00	0.20
	**D538G**	c.1613A > G	COSM94250	0.21	0.53
***AKT1***	**E17K**	c.49G > A	COSM33765	0.20	0.53

**Fig. 4:**
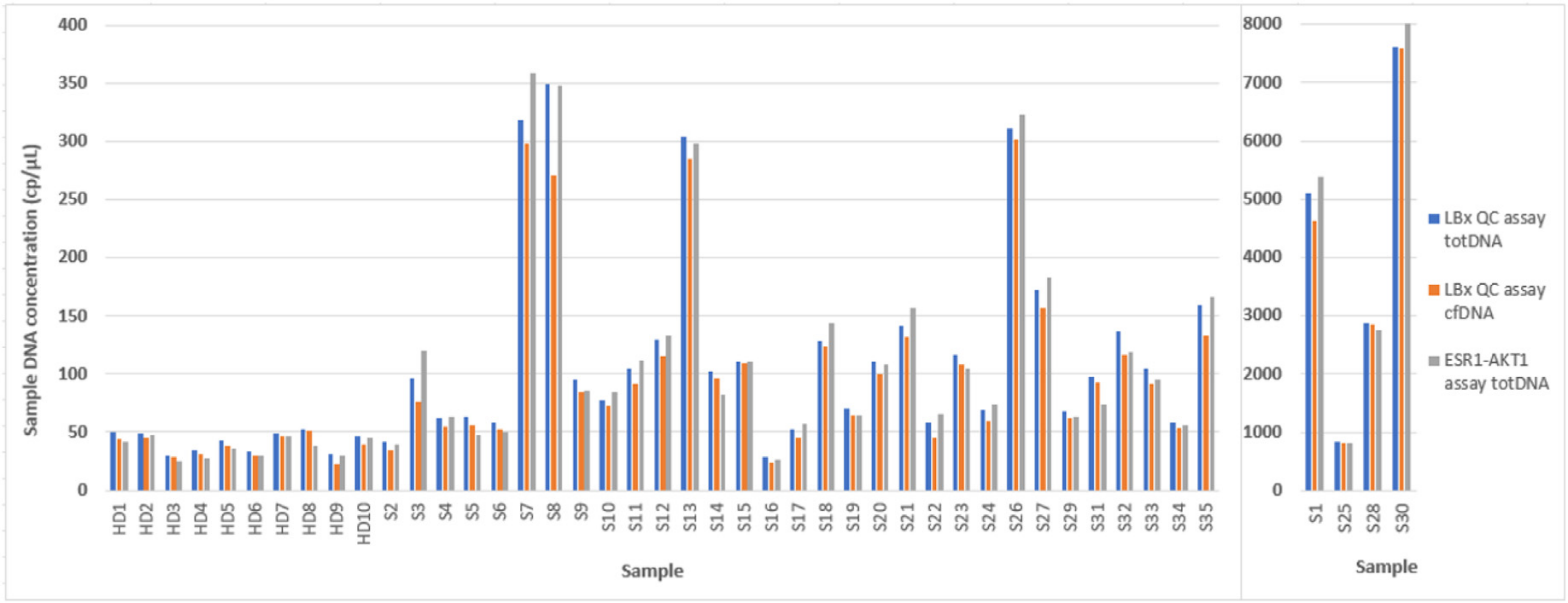
Comparison between plasma total DNA (blue) and cfDNA (orange) concentrations (copies/μl) based on the LBx QC-assay and total DNA concentrations (grey) based on the *TSN* reference gene of Stilla *ESR1-AKT* 6-color Crystal Digital PCR® assay. With the LBx QC assay quantifications, difference between total DNA (blue) and cfDNA (orange) represents the gDNA concentration. (For interpretation of the references to color in this figure legend, the reader is referred to the Web version of this article.)

The comparison of plasma-cfDNA concentrations measured with LBx QC assay and the Stilla *ESR1-AKT* 6-color Crystal Digital PCR® showed a good correlation (Fig. 4).

**Fig. 5:**
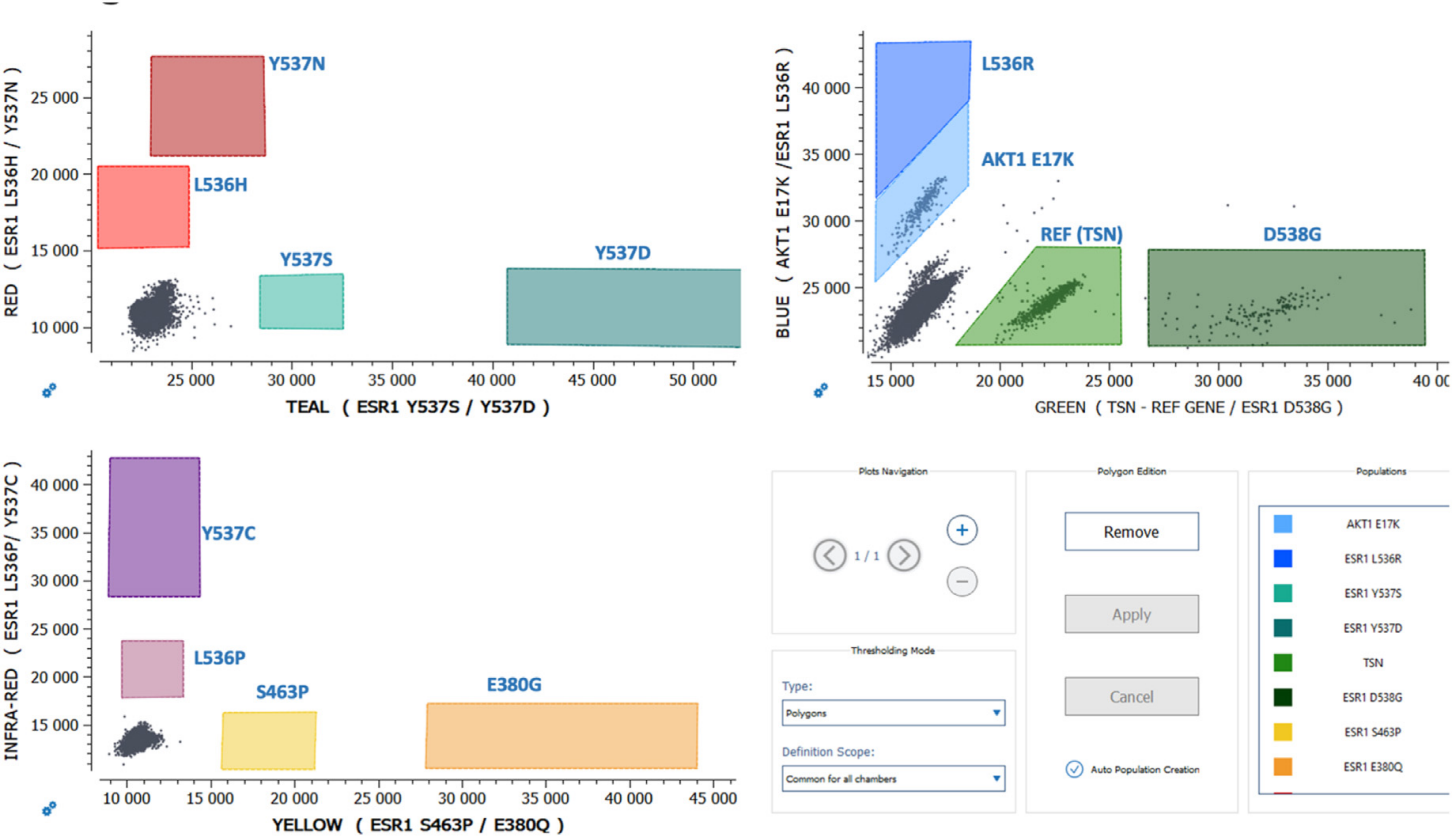
Characteristic 2D plot, illustrating the placement of the polygons of one plasma-cfDNA positive sample (S32) for *AKT1* E17K and *ESR1* D538G mutation with MAF of 37.0% and 25.3% respectively.

Using the Stilla *ESR1-AKT* 6-color Crystal Digital PCR® assay, all ten HD plasma-cfDNA samples were found negative for all *ESR1* mutations and *AKT1* E17K. Five of 35 plasma-cfDNA samples (14.3%) were found positive for both the *AKT1* E17K and *ESR1* D538G mutations (Fig. 5).

The MAF (%) in patient samples ranged from 1.1% to 37.0% for the *AKT1* E17K mutation and from 1.6% to 27.6% for the *ESR1* D538G mutation (Table 2).

**Table 2:** *ESR1* and *AKT* mutations detected in plasma-cfDNA (MAF%) of ER ​+ ​breast cancer patients with verified metastasis using Stilla *ESR1-AKT* 6-color Crystal Digital PCR® assay.

Sample code	Mutation detected	MAF (%)	Mutation detected	MAF (%)
S17	*AKT1* E17K	2.5	*ESR1* D538G	3.4
S19	*AKT1* E17K	36.1	*ESR1* D538G	27.6
S30	*AKT1* E17K	1.1	*ESR1* D538G	1.6
S31	*AKT1* E17K	23.2	*ESR1* D538G	13.9
S32	*AKT1* E17K	37.0	*ESR1* D538G	25.3

### 3.1 Direct comparison between Stilla *ESR1-AKT* 6-color crystal digital PCR® assay and the NAPA *ESR1* assay

We further directly compared the results obtained using the multiplex *ESR1-AKT* 6-color Crystal Digital PCR® assay with the *ESR1* NAPA assay (Table 3). The *ESR1* D538G mutation was detected in 4/35 (11.4%) plasma-cfDNA samples using the *ESR1* NAPA assay, while *ESR1* Y537S, *ESR1* Y537C and *ESR1* Y537N were not detected by either method in any of the plasma-cfDNA patient samples tested. No other *ESR1* mutations were detected in all clinical samples tested. Direct comparison between Crystal Digital PCR® and the *ESR1* NAPA assay based on Cohen's kappa coefficient (k) test revealed a high concordance (97.1%; k =0.87; p<0.001) for 34/35 samples tested, for the detection of the D538G mutation (Table 3). One sample was found positive using the Stilla *ESR1-AKT* 6-color Crystal Digital PCR® assay, but negative with the *ESR1* NAPA assay.

**Table 3:** Direct comparison between Stilla *ESR1-AKT* 6-color Crystal Digital PCR® assay and the NAPA *ESR1* assay for *ESR1* D538G, Y537S, Y537C and Y537 ​N mutations in plasma-cfDNA of 35 ​ER ​+ ​patient samples.

		6-color Crystal Digital PCR™			
**NAPA *ESR1* Assay**	D538G		+	–	Total
		+	4	0	4
		–	**1**	30	31
		Total	5	30	35
		Concordance: **34/35 (97,1%)** k = 0.871; **p < 0.001**			
	Y537S		+	–	Total
		+	0	0	0
		–	0	35	35
		Total	0	35	35
		Concordance: **35/35 (100%)** k = 1.00			
	Y537C		+	–	Total
		+	0	0	0
		–	0	35	35
		Total	0	35	35
		Concordance: **35/35 (100%)** k = 1.00			
	Y537 N		+	–	Total
		+	0	0	0
		–	0	35	35
		Total	0	35	35
		Concordance: **35/35 (100%)** k = 1.00			

We have in parallel analyzed 32/35 of these clinical samples with a previously published drop-off ddPCR for *ESR1* mutations [28], and the results were comparable, since we had a concordance of 100% (32/32); p<0.001.

## 4 Discussion

Clinicians' need for detecting specific subsets of genetic alterations is increasing as our understanding on resistance mechanisms to ET from patients with *ESR1* mutations progresses, and new therapies gets deeper [9,11,15,29]. It is now clear that plasma-cfDNA analysis to track *ESR1* mutations is highly beneficial for identifying tumor molecular dynamics and improving personalized treatments for mBC patients [30]. Based on plasma-cfDNA analysis, the safety and efficacy of palbociclib in combination with endocrine therapy, guided by *ESR1* mutation monitoring, has been evaluated in a randomized, open-label, multicentric phase III trial [31]. The PADA-1 trial has demonstrated the clinical efficacy of periodic monitoring for the emergence or rise of *ESR1* mutations in ctDNA. Such monitoring can prompt an early change from an aromatase inhibitor plus palbociclib to fulvestrant plus palbociclib treatment. Moreover, different molecular methods were used in the PADA-1 and EMERALD trials to evaluate the mutational status of *ESR1* in ctDNA, emphasizing its clinical importance [32,33]. Moreover, *ESR1* mutations detected in plasma-cfDNA are now a valuable tool for clinicians to determine the best course of treatment involving elacestrant (RAD-1901), a new type of selective estrogen receptor degrader (SERD) that was approved last year for the treatment of postmenopausal women and adult men with *ESR1*-mutated advanced or metastatic breast cancer who are ER+/HER2- and have had disease recurrence or progression [16,34]. Recently, a cohort study consisting of 3209 patients with different types of cancer at stage IV revealed that patients with advanced HR+ breast cancer undergoing concurrent testing had a higher detection rate of actionable variants that supports baseline ctDNA detection for serial monitoring of molecular evolution [35]. The detection of *ESR1* mutations through serial ctDNA monitoring is a hot topic now in the clinical field, since it allows for the quantitative assessment of changes in its allele frequency among patients who have advanced HR+ ​breast cancer as frontline treatment. The ability to monitor *ESR1* mutations through serial ctDNA testing holds great promise in improving patient outcomes by enabling clinicians to adjust treatment regimens in response to real-time changes in the tumor's genetic profile [36].

Crystal Digital PCR® has been successfully used up to now for various oncology applications such as copy number variation (CNV), mutation detection, rare event detection, and therapy monitoring [37,38]. This technology enables the precise quantification of multiple genetic targets in a single experiment. It is an easy-to-use digital PCR platform whose state-of-the-art microfluidic technology automatically integrates the digital PCR workflow into a single, ready-to-use chip. By increasing the multiplexing capabilities of digital PCR, we can decrease costs, increase throughput, and optimize the use of limited sample quantities. In order to ensure the accuracy and reliability of data obtained from digital PCR assays, higher multiplexing can be utilized. This allows for the inclusion of multiple control samples, which can be used to validate the accuracy of the results. By incorporating more controls within a single assay, the accuracy of the results can be further improved. Additionally, higher multiplexing digital PCR can help to reduce the time and cost associated with performing multiple separate assays, while also minimizing the risk of errors associated with the handling and processing of multiple samples.

A limitation of our study is the limited sample size, especially the small number of healthy controls. Future research to extend validation of this assay to a larger cohort of patients, incorporating samples from patients with more diverse treatment histories (e.g., endocrine therapy with aromatase inhibitors) could enrich the clinical relevance of our findings.

Overall, the results of this proof-of-principle study indicate that the Stilla *ESR1-AKT* 6-color Crystal Digital PCR® assay enables a fast and easy workflow for the detection and quantification of *ESR1* and *AKT1* hotspot mutations in plasma-cfDNA, saving a) precious liquid biopsy samples due to its multiplex design, and b) time since the whole procedure can be completed within 3h, with less than 10min of hands-on time.

## 5 Conclusions

In conclusion, our findings indicate that the Stilla *ESR1-AKT* 6-color Crystal Digital PCR® assay is reliable, robust, quantitative, highly sensitive and easy to perform in plasma-cfDNA samples. It is low cost, and it requires a relatively short time of analysis. Its high sensitivity makes it an ideal tool for reflex testing of the tumor in cases where a negative result is obtained in plasma-cfDNA analysis. The use of this assay provides an efficient and effective method for diagnosing cancer, and its ability to quickly and accurately detect tumor DNA in a patient's blood enables healthcare professionals to take timely and appropriate action. The clinical evaluation of this assay should follow, based on a larger number of clinical samples with well-defined clinical characteristics.

## Author contributions

Conceptualization, E.L.; methodology, S.S.; formal analysis, S.S. and E.L.; resources, V.G.; validation and clinical samples, S.S. and A.N.; data curation, S.S. and D.S.; writing—original draft preparation, S.S.; writing—review and editing, S.S. and E.L.; visualization, E.L.; supervision, E.L.; All authors have read and agreed to the submitted version of the current manuscript.

## Declaration of competing interest

The authors declare the following financial interests/personal relationships which may be considered as potential competing interests:

Evi Lianidou reports article publishing charges and equipment, drugs, or supplies were provided by STILLA TECHNOLOGIES. If there are other authors, they declare that they have no known competing financial interests or personal relationships that could have appeared to influence the work reported in this paper.
